# The Interaction between Canine Semen Bacteria and Semen Quality Parameters

**DOI:** 10.3390/ani14152151

**Published:** 2024-07-24

**Authors:** Šarūnė Sorkytė, Rita Šiugždinienė, Marius Virgailis, Gintarė Vaičiulienė, Anna Wysokińska, Ewa Wójcik, Paulius Matusevičius, Audronė Rekešiūtė, Neringa Sutkevičienė

**Affiliations:** 1 Animal Reproduction Laboratory, Faculty of Veterinary Medicine, Veterinary Academy, Lithuanian University of Health Sciences, Tilžės Str. 18, LT-47181 Kaunas, Lithuania; gintare.vaiciuliene@lsmu.lt (G.V.); audrone.rekesiute@lsmu.lt (A.R.); neringa.sutkeviciene@lsmu.lt (N.S.); 2 Institute of Microbiology and Virology, Faculty of Veterinary Medicine, Veterinary Academy, Lithuanian University of Health Sciences, Tilžės Str. 18, LT-47181 Kaunas, Lithuania; rita.siugzdiniene@lsmu.lt (R.Š.); marius.virgailis@lsmu.lt (M.V.); 3 Institute of Animal Science and Fisheries, Faculty of Agricultural Sciences, University of Siedlce, Konarskiego 2, 08110 Siedlce, Poland; anna.wysokinska@uws.edu.pl (A.W.); ewa.wojcik@uph.edu.pl (E.W.); 4 Department of Animal Nutrition, Faculty of Animal Sciences, Veterinary Academy, Lithuanian University of Health Sciences, Tilžės Str. 18, LT-47181 Kaunas, Lithuania; paulius.matusevicius@lsmu.lt

**Keywords:** canine, seminal bacteria, semen quality

## Abstract

**Simple Summary:**

In recent decades, the practice of artificial insemination has become increasingly prevalent in canine reproduction, thereby underscoring the necessity for improved semen quality assessment routines. While the bacterial microflora of the reproductive system of female dogs has been extensively studied, there is comparatively limited analysis of the male dog’s reproductive tract’s microbiota. In the field of andrology, a few connections have already been discovered between seminal bacteria and semen’s qualitative parameters. The objective of this study was to determine whether the bacteria present in semen samples from breeding dogs can influence the semen’s characteristics. The results indicated that multiple bacterial species present in semen samples from breeding dogs are associated with inferior sperm parameters. Moreover, a greater bacterial load was observed to be related with poorer semen quality in breeding dogs.

**Abstract:**

Assessing canine semen quality helps to detect infertility in males, but identifying factors that influence canine semen quality is a complicated task. The objective of this study was the assessment of the potential influence of bacteria found in canine semen samples on the characteristics of dogs’ semen. In this study, semen samples were collected manually from 30 dogs and subjected to a comprehensive examination. The results of sperm motility, concentration, viability, and morphology were statistically analysed in relation to the number of bacteria in the semen (CFUs/mL) and the seminal microbiota. Samples with an increased bacterial count per millilitre were associated with lower-quality sperm motility (*p* < 0.05). The most frequently isolated bacterial genera from the analysed semen samples were *Staphylococcus* spp. (26.0%), *Corynebacterium* spp. (17.8%), and *Streptococcus* spp. (16.4%). The presence of β-haemolytic *Escherichia coli* bacteria was linked to suboptimal semen samples, characterised by significantly reduced semen viability and a lower proportion of morphologically normal spermatozoa (*p* < 0.05). *Corynebacterium* spp. was associated with reduced bacterial load and superior semen quality (*p* < 0.01). These findings highlight the importance of bacterial cell counts and microbiota diversity in relation to various factors influencing canine semen quality, providing a more comprehensive understanding of canine reproductive well-being.

## 1. Introduction

The assessment of canine semen’s quality has become a standard procedure for the identification of infertility in dogs [1]. The evaluation of the semen’s quality determines the male’s fertility status and helps to identify semen that is suitable for artificial insemination (AI) and cryopreservation [2]. The assessment of semen’s quality parameters has become straightforward with the advent of modern technologies and advanced research methodologies [3]. Nevertheless, identifying the specific factors that influence variations in semen’s quality characteristics remains a challenging task.

The microbiota of animals can influence several physiological factors in organisms and affect their health [4]. It is noteworthy that studies investigating the microbiota in the male reproductive system of dogs and its influence on animal fertility are limited in number [4]. In contrast, research has predominantly focused on the microbiota of the bitch reproductive tract and its association with reproductive success [5]. However, seminal bacteria, which can be transferred to female dogs’ reproductive tracts, have the potential to trigger infections, spontaneous abortion, or even infertility [6,7].

It is well established that canine semen is not sterile, and various opportunistic bacteria have been detected within the male dog reproductive system [8,9]. The data presented in other articles indicate a correlation between the identified bacteria and semen quality across a range of animal species, including bulls, boars, poultry, etc. [4,10,11,12,13]. In the literature, some male genital tract bacteria are described as potentially more pathogenic. However, there are a lack of published data on the seminal microbiota, including which bacteria are commensal and which are pathogenic [14]. *Escherichia coli* is more frequently associated with less desirable semen quality [13]. A high bacterial load in semen (bacteriospermia) is related to lower sperm motility, membrane and acrosome integrity alterations, and higher oxidative stress. In canines, alterations in semen quality, including teratospermia, have been linked to specific pathogenic bacterial strains, such as β-haemolytic *Streptococcus* spp. [9]. Additionally, it has been observed that canine semen samples with bacteriospermia demonstrate lower sperm viability and a higher prevalence of morphological abnormalities in their spermatozoa [8].

As recent findings have highlighted the significant role of semen microbiota in male infertility, it is becoming increasingly important to extend bacteriological studies to include a wider variety of animal species. Acknowledging that canine semen contains bacterial populations indicates the possibility that specific bacteria may impact semen quality characteristics, particularly its motility, which is closely associated with reproductive success in canines [15]. Therefore, the aim of this study was to evaluate the impact of bacteria in the semen of breeding dogs on the semen’s quality.

## 2. Materials and Methods

### 2.1. Animals

Semen samples were obtained from 30 clinically healthy canines (*Canis familiaris*). The dogs exhibited a range of sizes, with the following breeds represented: German Shepherds (n = 5), Labrador Retrievers (n = 3), Salukis (n = 2), Bull Terriers (n = 2), Shih Tzus (n = 3), and several other breeds. It was confirmed that the animals in question had not received any antibiotics within the preceding month. The collection of canine semen and the external examination of the canines were conducted in accordance with EU Directive 2010/63/EU for animal experimentation.

### 2.2. Semen Collection and Assessment

The samples were collected at a veterinary clinic. Prior to semen collection, the clinical condition of each dog was assessed. An external examination of the dog’s reproductive system was conducted to identify any signs of pain or discomfort in the reproductive organs, including signs of inflammation [1]. The detection of inflammation in dogs was done through palpation, which involved the detection of symptoms such as swelling, redness, heat, and pain. The sample collection procedure was conducted in the presence of a female in oestrus or with the use of swabs containing vaginal secretions from a female dog in oestrus. Sperm-rich fractions were collected into sterile plastic bags through digital manipulation, as previously described [16]. To prevent bacterial contamination from the penile prepuce, the tip of the glans penis was protruded from the prepuce and cleaned with sterile gauze before collection. Samples of semen for bacteriological investigation were obtained immediately after semen collection with a pipette using sterile tips. Volumes of 0.2 mL of semen were transferred into sterile 1.5 mL Snap Cap Microcentrifuge Tubes (Thermo Fisher Scientific, Hennigsdorf, Germany) and transported to the microbiology laboratory within a 15–30 min period in an insulated box with a cold pack. Semen samples, for the assessment of their quality, were delivered to the laboratory in a thermos with a temperature of 35 ± 2 °C within 30 min.

### 2.3. Semen Quality Assessment

The evaluation of semen quality was performed at the Laboratory of Animal Reproduction of the Lithuanian University of Health Sciences. The following parameters were analysed: semen volume, pH, sperm motility, concentration, viability, and morphological changes of spermatozoa.

The most important semen quality parameter, motility, was assessed subjectively immediately after sample collection: 10 µL of the semen sample were placed on a microscope slide (Avantor VWR^®^, Radnor, PA, USA), covered with a coverslip, and placed on a heated plate at 37 °C. The slide was then observed under an Eclipse 50i microscope (Nikon^®^, Tokyo, Japan).

The pH of the samples was measured using an electronic pH meter, the Fisherbrand accumet AB150 (Fisher scientific, Loughborough, UK). The concentration of semen was determined using an improved neubauer haemocytometer chamber (BLAUBRAND^®^, Wertheim, Germany) and the results were expressed as the number of spermatozoa per millilitre. The total number of sperm present in the ejaculate was calculated by multiplying the concentration per millilitre by the volume.

Semen viability was assessed through smears of the samples, which were stained with the following eosin–nigrosin dyes: Eosin G stain, 2% solution, and Nigrosin stain, 4% solution for live/dead cells (Minitube, Tiefenbach, Germany). A drop of the semen sample was placed on a slide, followed by a drop of eosin–nigrosin dye, which was then mixed. After half a minute, a smear was made, the slide was air-dried, and a microscopic evaluation was performed, assessing the staining of 100 spermatozoa. In accordance with the test principles employed, the unstained sperm were considered to be viable.

For the assessment of morphological abnormalities in the spermatozoa, a semen sample smear was stained with the SpermBlue staining kit (Microptic SL, Barcelona, Spain) in accordance with the procedure of van der Horst and Maree [17]. A total of 500 sperm were evaluated under a light microscope, the Eclipse 50i, with an oil immersion objective at 100× magnification. The following abnormalities were included in the sperm head morphological defects: macrocephaly, microcephaly, tapered head, thin, narrow head, pinhead, undeveloped sperm, and asymmetrical midpiece insertion into the head. Abnormalities of the tail included irregularities of the midpiece (e.g., a bent neck, a neck that is too thick or too thin, or an irregularly shaped neck), a coiled tail, a stump tail, and a duplicate tail. Other morphological defects encompassed droplets, acrosome defects, vacuoles, or absent tails (tailless). The information on the spermatozoa’s morphology was systematically recorded, including head, tail, and other abnormalities, in the prepared data tables. Additionally, the number of morphologically normal spermatozoa was recorded.

### 2.4. Bacteriological Analysis of Semen

The bacteriological analysis of the semen samples was conducted at the Lithuanian University of Health Sciences, Institute of Microbiology and Virology. In brief, 10 µL of each sample was inoculated on Columbia Agar plates with 5% Sheep Blood (E&O Laboratories Ltd., Bonnybridge, Scotland, UK) using serial dilutions. The inoculated plates were incubated for 24–48 h at 37 °C under aerobic conditions. After incubation, the number of colony-forming units (CFUs) per millilitre were recorded.

The bacteria colonies were collected for further investigation. For further identification, pure bacterial cultures were inoculated onto selective agars. The morphology of the bacteria was examined by Gram staining, and the genera and families were identified by biochemical properties, including the catalase test [18,19]. The bacteria belonging to the *Staphylococcus* genus were identified to the species level using the “RapID™ STAPH PLUS System” identification systems (Thermo Fisher Scientific, Hennigsdorf, Germany) and associated computer program according to the instructions. Gram-negative bacteria were identified using the “Novacyt–Microgen Bioproducts GN-ID A+B-Combined 30 Test System” identification systems (Thermo Fisher Scientific, Hennigsdorf, Germany), along with the corresponding computer program and according to the manufacturer’s instructions.

Bacteria that could not be identified under laboratory conditions were sent to the Lithuanian National Food and Veterinary Risk Assessment Institute for MALDI-TOF MS (matrix-assisted laser desorption ionization–time of flight Mass Spectrometry) (Bruker Daltonics, Bremen, Germany) biotyping.

### 2.5. Sample Grouping

The research subjects were grouped into three size groups: small dogs (<10 kg), n = 10; medium dogs (10–30 kg), n = 11; and large dogs (>30 kg), n = 9.

Semen samples were divided into 3 high- and 3 low-quality groups (H and L) by qualitative criteria, according to motility, viability, and morphology (Table 1). The morphology was based on the percentage of normal morphology form spermatozoa (normal spermatozoa). Samples that met all of the pre-established criteria were included in the high-quality (H QUALITY) group, while those samples that failed to meet at least one of the criteria were included in the low-quality (L QUALITY) group.

### 2.6. Data Analysis

The statistical data analysis was performed using “IBM SPSS Statistics 29.0.0.0”. Descriptive statistics were calculated, and statistical tests, including the Student’s *t*-test, Mann–Whitney U test, and Pearson’s correlation, were used to assess differences and correlations among the samples’ bacterial numbers, variable aerobic bacteria isolations, and different dog semen quality parameters. Because the normal distribution was not always fulfilled, non-parametric statistical analysis was performed. The data are presented as means (standard deviation (SD)). Results were considered statistically significant when the *p*-value < 0.05. Exact *p*-values are provided for all comparisons, except when *p* is below 0.001 (*p* < 0.001).

## 3. Results

Bacterial populations were detected in all 30 canine semen samples. In these samples, a total of 11 different bacterial families were identified. The bacterial genera associated with each family are presented in Table 2. The most frequently isolated bacterial genera from the analysed semen samples were *Staphylococcus* spp. (26.0%), *Corynebacterium* spp. (17.8%), and *Streptococcus* spp. (16.4%). The most predominantly isolated bacterial species from the *Staphylococcus* spp. genus were *S. pseudintermedius* (31.6%), *S. epidermidis* (15.8%), and *S. aureus* (15.8%). Additionally, *S. schleiferi* and *S. intermedius* were isolated, while other staphylococci species were identified but not at the species level. *Corynebacterium imitans* was the only species that could be identified from the *Corynebacterium* spp. genera with the system used. The bacterium *Streptococcus canis* constituted 50 percent of all identified streptococci, with the remaining half consisting of *S. sanguinis* and other streptococci. From *Neisseria* spp., the only species present was *Neisseria weaveri*. *Acinetobacter* spp. consisted entirely of the species *Acinetobacter haemolyticus*. Of the *Micrococcus* spp. isolates, only one was identified to the species level, as *Micrococcus luteus*, while the remainder remained unidentified at the species level. Both bacteria from the *Escherichia* spp. genus were identified as *Escherichia coli* (β-haemolytic). All bacteria from the families *Pasteurellaceae*, *Microbacteriaceae*, and *Lactobacillaceae* were identified to the species level. These included *Haemophilus* spp.—*Haemophilus haemoglobinophilus*, *Frederiksenia* spp.—*Frederiksenia canicolla*, *Pasteurella* spp.—*Pasteurella multocida*, *Canibacter* spp.—*Canibacter oris*, and *Limosilactobacillus* spp.—*Limosilactobacillus reuteri*.

The bacterial count in the canine semen samples was not found to be significantly affected by the size (weight) of the dog (*p* = 0.347).

**Figure 1:**
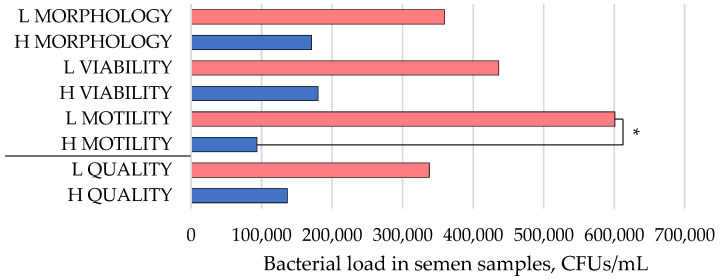
A diagram illustrating the bacterial contamination in semen samples, expressed as colony-forming units (CFUs) per millilitre of sample. Semen samples are classified into higher (H, blue colour) and lower (L, red colour) quality groups according to their quality parameters: viability, morphology, and motility. H VIABILITY represents samples with 50% viability and higher, while L VIABILITY represents samples with less than 50% viability. H MORPHOLOGY indicates samples with 70% morphologically normal spermatozoa and higher, and L MORPHOLOGY represents samples with less than 70% morphologically normal spermatozoa. H MOTILITY designates samples with motility higher than 75%, and L MOTILITY represents samples with 75% motility and lower. H QUALITY and L QUALITY represent general quality groups. * Significant differences between groups when *p*-value < 0.05.

The bacterial contamination results for the canine semen samples were determined according to the categorisations of motility, viability, and morphology, as presented in Table 1. These results are displayed in Figure 1. In samples exhibiting optimal viability and morphological quality (blue colour), a lower bacterial number was observed, although this was not statistically significant (*p* > 0.05). Samples with lower motility showed a statistically significantly higher number of bacteria in their semen (*p* = 0.035) (Figure 1).

**Figure 2:**
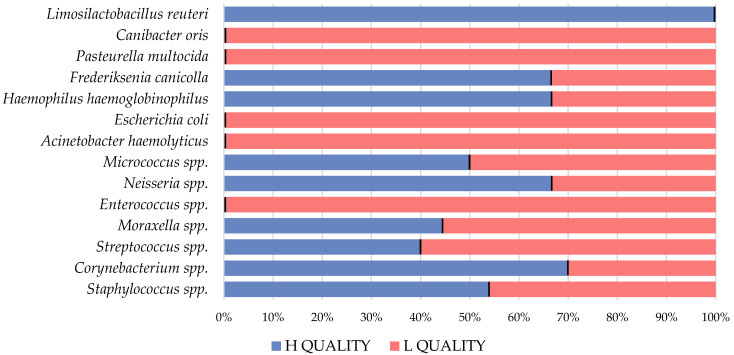
A diagram illustrating the percentage distribution of identified bacteria in semen samples of two quality groups, according to their viability, motility, and morphology.

The association between different bacteria and the quality of the examined semen samples was evaluated by monitoring the frequencies of detection of different bacteria in samples of high and low semen quality (Figure 2). *Limosilactobacillus reuteri* was identified only in one optimal-quality semen sample. The isolation of *Corynebacterium* spp., *Neisseria* spp., *Frederiksenia canicolla*, and *Haemophilus haemoglobinophilus* was observed to occur with greater frequency in superior quality semen samples. *Canibacter oris*, *Pasteurella multocida*, *E. coli*, *A. haemolyticus,* and *Enterococcus* spp. were only detected in samples of inferior semen quality.

The means of the parameters, including motility, viability, and detailed semen morphology, are presented in Table 3 by dividing the samples according to the presence or absence of bacteria. Samples with each detected bacterium were compared with samples where that bacterium was not detected. Several notable differences were observed. Samples containing β-haemolytic *E. coli* were associated with inferior sperm viability (*p* = 0.03) and a lower number of morphologically normal spermatozoa (*p* = 0.02). Conversely, semen samples in which *Corynebacterium* spp. were isolated had a significantly reduced number of spermatozoa other abnormalities (*p* = 0.045), while sperm motility was significantly higher than in other samples without this bacterium (*p* = 0.006). Moreover, the bacterial count was found to be significantly lower in these samples (*p* = 0.028).

## 4. Discussion

The results of our study demonstrated that none of the canine semen samples were sterile, and 11 distinct bacterial families were identified. In the analysis of 30 healthy canine semen samples, the most frequently detected bacteria were identified as *Staphylococcus* spp. (26.0%), *Corynebacterium* spp. (17.8%), and *Streptococcus* spp. (16.4%). In smaller quantities, the *Moraxella* spp., *Enterococcus* spp., *Pastuarellaceae*, *Neisseria* spp., *Microbacteriaceae*, *Acinetobacter haemolyticus*, *E. coli*, *Canibacter oris*, and *Lactobacillus reuteri* families, genera, and species were detected. In 2022, a related study conducted by S. Agudelo-Yepes et al. revealed relevant results about nonsterile dog semen samples. The study demonstrated that bacterial populations were present in 100% of the examined dog semen samples [8].

In the previously mentioned study on the microbiota of canine semen, the presence of the following bacteria was detected in healthy canine semen samples: *Staphylococcus* spp., *Streptococcus* spp., *E. coli*, *Klebsiella* spp., *Neisseria gonorrhoeae*, *Pseudomonas* spp., and *Chlamydia trachomatis* [8]. Other research has revealed a broader variety of bacteria in canine semen [9]. However, none of these studies identified the presence of *Canibacter oris*. *C. oris* bacteria are strongly associated with the canine oral bacterial microflora and are more frequently described as pathogens in dog bite wounds [20]. Consequently, the detection of this bacterium in semen indicates that the oral microbiota of dogs can enter the canine reproductive tract, and bacteria from the mouth can be identified in the seminal microbiota of dogs. Knowing that bacteria can enter a dog’s reproductive tract from its mouth, it is possible that bacteria can also travel in the opposite direction, from the male reproductive tract to the oral cavity. Scientists have observed that several bacterial species may be transmitted from the oral flora of dogs to their owners through close physical contact, as described in this article [21]. Overall, there is a possibility that pathogenic bacteria discovered in the male dog reproductive system could, over time, be transported to the dog’s oral flora and subsequently transmitted as zoonotic bacteria to the dog owner.

In our research, samples with a higher number of bacteria were associated with lower motility in semen samples. A previous study conducted at the Columbian university (Universidad de Antioquia) has discovered related results. The study found that bacteriospermia in dog semen negatively affect sperm motility and spermatozoa morphology [8]. The negative influence of seminal bacteria can be explained as follows: several bacteria can directly attach to spermatozoa, immobilise them, and induce agglutination, autoimmune reactions, or oxidative stress, and some bacteria may even excrete toxins [4,22]. The present study aimed to analyse the effects of different bacteria on the parameters of semen quality. The results revealed significant differences. In the present study, potentially pathogenic seminal bacteria previously associated with poor semen quality in other species were detected. Samples containing β-haemolytic *Escherichia coli* exhibited reduced viability and a greater prevalence of other sperm morphological pathologies. To the best of our knowledge, this bacterium has not previously been associated with changes in the quality parameters of canine semen. However, related results have been observed in other animal species research, where *E. coli* was associated with inferior semen samples of boars [23,24]. In our study, for dog semen samples with β-haemolytic *E. coli*, the average viability was two times lower than in samples without this bacterium. This could be attributed to the α-haemolysin produced by *E. coli*, which is a potent cytotoxin capable of damaging the integrity of the sperm membrane and forming pores in the cell membrane [22]. In an earlier study, a statistically significant negative correlation was observed between the presence of *E. coli* in semen used for gilt insemination and litter size [24]. Nevertheless, studies comparing the microbiota of dog semen with the number of offspring produced by females, according to our knowledge, have not yet been conducted.

In microbiology field, some authors claim that a few strains of *Staphylococcus* spp. can produce substances believed to be like the staphylococcal enterotoxins SAF (sperm agglutination factor) and SIF (sperm immobilization factor), which can immobilize sperm and promote their agglutination [22]. This could be a contributing factor to the decrease in sample motility observed with *Staphylococcus* spp. in our research. *Enterococcus* spp. is described as secreting the toxin β-haemolysin, which also has the ability to immobilize sperm [25]. Additionally, some bacteria can form biofilms, which induce sperm agglutination and decreases motility [22].

In our study, samples in which bacteria from the *Corynebacterium* spp., *Neisseria* spp., and *Streptococcus* spp. genera or the bacterium *L. reuteri* were identified had a higher motility than samples in which these bacteria were not detected. A statistically significant difference was observed for *Corynebacterium* spp. Additionally, in samples where *Corynebacterium* spp. was isolated, statistically significantly fewer other abnormalities were found in comparison to samples without this bacterium. Moreover, these samples were associated with lower bacterial counts in semen. Thereby, the assumption can be made that possibly the *Corynebacterium* spp. found in our study samples can inhibit the proliferation of other microbes. The results indicate that bacteria belonging to the *Corynebacterium* spp., *Neisseria* spp., *Streptococcus* spp., and *Lactobacillus* spp. genera, found in this research, do not negatively impact sperm motility. It can be assumed that these bacteria represent a normal component of the seminal microflora. The presence of such bacteria may be indicative of the absence of competing species that have a more negative effect on semen. It can be hypothesized that these bacteria may act as commensals, protecting the dog’s semen from invasion by more pathogenic bacteria.

In our research, *Limosilactobacillus reuteri* was found to be associated with an excellent-quality semen sample. However, due to the fact that this bacterium was identified in only one semen sample, the result was not statistically significant. In other articles, the bacterium *L. reuteri* is described as a probiotic strain capable of excreting antimicrobial molecules, inhibiting the growth of pathogenic microbes, and having a strong anti-inflammatory effect. As previously demonstrated, the administration of *L. reuteri* as an oral supplement to canines has the potential to enhance the qualitative parameters of semen [26]. It can be considered that a similar mechanism of action is present in canine semen, whereby the bacterium directly reduces inflammation or the growth of pathogenic microbes. Nevertheless, further in vitro studies are required to confirm this assumption.

## 5. Conclusions

It has been demonstrated that bacteriospermia has a significant impact on the quality of canine semen. This is evidenced by an association between a higher number of bacteria (CFUs) in semen and decreased seminal qualitative parameters. It is important to note that not all bacteria present in canine semen have a detrimental impact on its quality. Rather, different bacteria may be associated with alterations in distinct qualitative parameters. A number of bacterial species, including *Corynebacterium* spp., *Neisseria* spp., *Streptococcus* spp., and *Limosilactobacillus reuteri*, were not found to have a negative impact on semen quality. In contrast, the samples with *Corynebacterium* spp. were linked to a reduced bacterial load, which also resulted in superior qualitative parameters. However, other bacteria were more frequently associated with inferior semen quality. Of particular interest in this study was the identification of β-haemolytic *Escherichia coli* as the most pathogenic bacteria for canine semen parameters.

## Figures and Tables

**Table 1:** Canine semen sample grouping criteria.

Parameter	Superior Quality Semen (H); n = 10	Inferior Quality Semen (L); n = 20
Sperm motility (%)	>75%	≤75%
Live spermatozoa (%)	≥50%	<50%
Normal spermatozoa (%)	≥70%	<70%

**Table 2:** Bacterial families and genera isolated and identified from canine semen samples.

Identified Bacteria Families	Identified Bacteria Genus	Positive Semen Samples	
		N	Percentage ^1^, %
*Staphylococcaceae*	*Staphylococcus* spp.	19	63.3
*Corynebacteriaceae*	*Corynebacterium* spp.	13	43.3
*Streptococcaceae*	*Streptococcus* spp.	12	40.0
*Moraxellaceae*	*Moraxella* spp.	5	16.7
	*Acinetobacter* spp.	3	10.0
*Enterococcaceae*	*Enterococcus* spp.	5	16.7
*Neisseriaceae*	*Neisseria* spp.	4	13.3
*Micrococcaceae*	*Micrococcus* spp.	3	10.0
*Enterobacteriaceae*	*Escherichia* spp.	2	6.7
*Pasteurellaceae*	*Haemophilus* spp.	2	6.7
	*Frederiksenia* spp.	2	6.7
	*Pasteurella* spp.	1	3.3
*Microbacteriaceae*	*Canibacter* spp.	1	3.3
*Lactobacillaceae*	*Limosilactobacillus* spp.	1	3.3

^1^ The proportion of semen samples in which bacteria were identified.

**Table 3:** Comparison of semen quality parameters between samples where specific bacteria were identified and samples where those bacteria were not identified.

Identified Bacteria (Genus/Species)	In Samples	Motility, %	Viability, %	Head Pathologies, %	Tail Pathologies, %	Other Pathologies, %	CFUs per mL, 10^5^
*Staphylococcus* spp.	Identified	66.39 (31.98)	55.35 (22.28)	7.77 (6.79)	14.53 (13.48)	19.97 (22.98)	2.52 (4.06)
	Not identified	79.55 (11.50)	52.36 (22.61)	8.64 (3.56)	8.09 (4.04)	24.59 (23.47)	2.76 (4.65)
*Streptococcus* spp.	Identified	76.67 (19.92)	52.91 (18.03)	7.35 (4.84)	13.38 (13.02)	20.21 (22.86)	1.85 (3.84)
	Not identified	67.65 (30.57)	55.00 (24.79)	8.62 (6.36)	11.18 (10.12)	22.79 (23.50)	3.11 (4.47)
*Corynebacterium* spp.	Identified	** 83.46 (4.74)	51.85 (25.52)	6.08 (2.90)	8.04 (4.86)	* 12.39 (13.76)	* 0.44 (0.96)
	Not identified	** 60.94 (32.52)	50.38 (22.79)	9.74 (6.92)	15.38 (13.83)	* 29.31 (26.21)	* 4.27 (4.96)
*Escherichia coli* (β-haemolytic)	Identified	75.00 (7.07)	* 21.50 (2.12)	6.10 (4.10)	6.00 (7.78)	* 70.75 (0.35)	5.05 (6.99)
	Not identified	70.74 (27.52)	* 55.33 (21.58)	8.24 (5.85)	12.54 (11.43)	* 18.09 (19.08)	2.43 (4.09)
*Enterococcus* spp.	Identified	51.25 (42.11)	49.00 (12.65)	10.45 (5.66)	17.25 (15.84)	26.00 (15.53)	2.44 (4.29)
	Not identified	74.60 (23.00)	53.64 (23.88)	7.72 (5.75)	11.26 (10.53)	21.04 (24.00)	2.64 (4.28)
*Moraxella* spp.	Identified	57.00 (43.53)	40.20 (19.61)	12.60 (11.35)	14.70 (14.83)	31.90 (29.08)	* 6.35 (5.03)
	Not identified	74.38 (21.93)	55.67 (22.57)	7.16 (3.48)	11.54 (10.66)	19.60 (21.49)	* 1.86 (3.67)
*Acinetobacter haemolyticus*	Identified	38.33 (40.72)	30.67 (7.77)	12.13 (5.33)	31.17 (22.43)	21.50 (16.89)	3.43 (5.69)
	Not identified	75.19 (22.74)	55.58 (22.31)	7.63 (5.67)	9.89 (7.25)	21.75 (23.72)	2.52 (4.14)
*Limosilactobacillus reuteri*	Identified	90.00 (0.00)	74.00 (0.00)	3.40 (0.00)	5.50 (0.00)	2.50 (0.00)	0.01 (0.00)
	Not identified	70.71 (26.90)	52.25 (22.59)	8.26 (5.75)	12.32 (11.37)	22.41 (22.97)	2.70 (4.25)
*Neisseria* spp.	Identified	85.00 (4.08)	58.50 (29.24)	4.80 (3.07)	7.88 (6.55)	27.00 (32.17)	0.03 (0.045)
	Not identified	69.20 (28.13)	52.12 (21.92)	8.62 (5.90)	12.76 (11.77)	20.88 (21.79)	3.01 (4.39)
*Micrococcus* spp.	Identified	61.67 (44.81)	37.33 (13.05)	9.40 (0.87)	23.33 (20.50)	16.50 (12.99)	4.98 (0.82)
	Not identified	72.50 (24.95)	54.81 (22.87)	7.95 (6.03)	10.79 (9.54)	22.33 (23.84)	2.84 (4.37)

The data are represented as means (SD). * Significant differences between groups when *p*-value < 0.05; ** *p*-value < 0.01.

## Data Availability

The data presented in this study are available within the article.
